# 4G/5G polymorphism and plasma levels of plasminogen activator inhibitor-1 in essential thrombocythemia patients with driver mutational status

**DOI:** 10.1186/s12959-026-00828-x

**Published:** 2026-01-17

**Authors:** Xueya Zhang, Xizhe Guo

**Affiliations:** Department of Hematology, The Second Affiliated Hospital of Fujian Medical University, 34 Zhongshan North Road, Quanzhou, Fujian Province 362000 China

**Keywords:** Essential thrombocythemia, Plasminogen activator inhibitor-1, 4G/5G gene polymorphism, JAK2 V617F mutation, Thrombosis

## Abstract

Essential thrombocythemia (ET) is a kind of myeloproliferative neoplasms. Thrombosis is one of the common symptoms, however, the incidence of thrombosis in ET with *JAK2*,* CALR*, *MPL* gene mutations varies greatly, and the underlying mechanism is unclear. The clinical data of 155 consecutive patients with ET were analyzed retrospectively. The plasminogen activator inhibitor-1 (PAI-1) 4G/5G polymorphism and plasma levels were measured by sequencing reaction universal kit and enzyme linked immunosorbent assay (ELISA), respectively. The mutational status in ET was detected by polymerase chain reaction (PCR). Thrombotic events were detected by Color Doppler Ultrasound, Computer Tomography (CT), Magnetic Resonance Imaging (MRI), angiography and D-dimer. Among 155 patients with ET, 21.3% (33/155) had thrombotic events, including 54.5% (18/33) of ischemic stroke, 36.4% (12/33) of ischemic heart disease and 9.1% (3/33) of venous thrombosis. Compared to the healthy control population, there was no significant difference between the occurrence of thrombotic events and the patient’s age, gender, and blood cell counts, except for platelet counts. The incidence of thrombotic events in *JAK2 V617F* mutation positive ET patients was significantly higher than that in *CALR* mutation positive and *JAK2 V617F* mutation negative ET patients (*P* < 0.05). The gene 4G4G polymorphism and plasma levels of PAI-1 in *JAK2 V617F* mutated ET patients were significantly higher than that in *CALR* mutated ET patients, respectively (*P* < 0.05). In patients with *JAK2 V617F* mutation positive ET, the incidence of thrombosis in 4G4G gene group and plasma levels of PAI-1 group were significantly higher than that in non 4G4G group, respectively (*P* < 0.05). The 4G4G gene polymorphism and high plasma levels of PAI-1 have potential value in predicting the risk factors for thrombosis in *JAK2 V617F* mutation positive ET compare to *CALR* mutation positive subjects.

## Introduction

Essential thrombocythemia (ET) is a clonal proliferative disease of hematopoietic stem cells. It is an independent subtype of classic myeloproliferative neoplasms. Its characteristics are abnormal increase of platelet counts in peripheral blood and significant proliferation of megakaryocytes in bone marrow [1–3]. Mutations of *JAK2 V617F*, thrombopoietin receptor (*MPL*), calreticulin (*CALR*) and other genes were detected in 97% of ET patients. Previous results showed that ET was often accompanied by thrombosis, hemorrhage, hepatosplenomegaly, transformation to bone marrow fibrosis, bone marrow failure, and acute leukemia [4–9]. At present, the study on thrombosis in ET patients is still unclear, and the risk of thrombosis in ET patients with positive *JAK2 V617F* mutation is significantly higher than that in those with positive *CALR* mutation, but the underlying mechanism is also still unclear [10, 11]. We retrospectively analyzed the clinical data of 155 consecutive ET patients, and found that Plasminogen activator inhibitor-1 (PAI-1) gene polymorphism, plasma levels of PAI-1 and *JAK2 V617F* mutation were independent risk factors for thrombotic events in ET patients. The 4G4G type of PAI-1 gene and plasma levels of PAI-1 were significantly higher in *JAK2 V617F* mutation positive ET patients than in *CALR* mutation positive ET patients.

## Materials and methods

### Patients information

From January 1, 2017 to December 31, 2024, we collected 155 consecutive ET patients who were hospitalized and outpatients in the Department of Hematology, the Second Affiliated Hospital of Fujian Medical University, including 75 male patients and 80 female patients, with median age of 60 years (22–90 years), all of whom met the WHO diagnostic criteria for MPN in 2016 [2]. All the participants signed the informed consent form. This study complied with Declaration of Helsinki. All bone marrow samples were collected with informed consent, and the study was reviewed and approved by the Institutional Review Board of the Second Affiliated Hospital of Fujian Medical University.

The basic data collected include: (1) General data: gender, age, blood routine examination; (2) Clinical manifestations: whether there are thrombosis events (including coronary heart disease, cerebral infarction and venous thrombosis); (3) Myeloproliferative neoplasms (MPNs) driver gene (*JAK2 V617F*,* CALR* exon 9, *MPL W515L/K*) mutation; (4) 4G/5G polymorphism of PAI-1 gene (4G4G, 4G5G, 5G5G).

### Detection of the differences among ET driven gene mutation

In order to detect driver gene mutations. Of all, 4 to 5 mL of bone marrow of all recruited patients were collected. The mutation types of *JAK2 V617F*,* MPL W515L/K* and *CALR* exon 9 gene were detected by polymerase chain reaction (PCR) and Sanger sequencing as previously described, respectively [5].

### Detection of 4G/5G gene polymorphism and plasma levels of PAI-1

The 4G/5G gene polymorphism and plasma levels of PAI-1 were measured by using sequencing reaction kit (Beijing Huaxia times Gene Technology Development Co., Ltd) and enzyme-linked immunosorbent assay (ELISA) kit (CUSABIO Biotechnology Co. LTD, Wuhan, China), respectively.

### Treatment and follow-up

According to the ET risk stratification, treatment with aspirin combined or not combined with hydroxyurea tablets should be administered. Within 3 months, follow up and check blood routine once a week. For patients with thrombosis, add D-dimer testing; Within 3–6 months, conduct a blood routine check every 2 weeks; Within 6–12 months, blood routine check every 4 weeks; Within 1–2 years, follow up with a blood routine examination every 2 months; Blood routine test every 3 months for more than 2 years.

### Statistical analysis

SPSS 28.0 statistical software was used to analyze and process the data. The measurement data were represented by X ± S, and independent sample t-test was used for comparison. The counting data were expressed by frequency and percentage (%), and the comparison was made by *χ*^2^ test and Fisher exact probability method. *P <* 0.05 means the difference is statistically significant.

## Results

Among 155 ET patients, 21.3% (33/155) had thrombotic events, including 54.5% (18/33) of ischemic stroke, 36.4% (12/33) of ischemic heart disease, and 9.1% (3/33) of venous thrombosis. Compared to the healthy control population, there was no significant difference between the occurrence of thrombotic events and the patient’s age, gender, and blood cell counts, except for platelet counts (Table 1). Among ET patients, the average age of thrombotic events was 61.67 ± 8.27, 20 males and 13 females; The average age of non thrombotic events was 59.57 ± 16.01, 55 males and 67 females. There was no significant difference in gender and age in thrombotic events (*P* > 0.05) (Table 2).

**Table 1:** Correlation analysis of clinical data and thrombotic events of patients with essential thrombocythemia and healthy control population

Clinical data		Essential thrombocythemia	Healthy control population	Value	*P*
Age (y)		60.85 ± 12.99	59.67 ± 11.53	0.45	0.65
Gender	male	75(48.4%)	63(48.1%)	0.01	0.92
	female	80(51.6%)	68(51.9%)	--	--
PAI-1 gene polymorphism	4G4G	40(25.8%)	46(35.1%)	4.82	0.09
	4G5G	60(38.7%)	51(38.9%)	0.76	0.38
	5G5G	55(35.5%)	34(26%)	1.25	0.26
Blood cells	WBC (×10^9^/L)	14.7 ± 7.35	9.8 ± 3.3	0.97	0.32
	HGB (g/L)	133.3 ± 17.8	121.6 ± 16.7	3.04	0.08
	PLT (×10^9^/L)	950 ± 320.6	306 ± 108.3	8.38	0.001
Cardiovascular risk factors	hypertension	32	26	0.96	0.33
	diabetes	19	13	0.29	0.59
	hyperlipidemia	13	15	0.54	0.46
	smoking history	37	29	0.29	0.59

**Table 2:** Correlation analysis of clinical data and thrombotic events of patients with essential thrombocythemia

Clinical data		Thrombotic events		Value	*P*
		With (33)	Without (122)		
Age (y)		61.67 ± 8.27	59.57 ± 16.01	0.06	0.81
Gender	male(75)	20(26.7%)	55(73.3%)	0.21	0.65
	female(80)	13(16.3%)	67(83.7%)	---	---
PAI-1 gene polymorphism	4G4G	18(54.5%)	22(18%)	118.23	<0.001
	4G5G	11(33.3%)	49(40.2%)	1.87	--
	5G5G	4(12.2%)	41(33.6%)	8.53	--
Blood cells	WBC (×10^9^/L)	16.5 ± 7.6	12.9 ± 7.1	2.16	0.39
	HGB (g/L)	136.9 ± 16.3	129.7 ± 19.2	51.33	0.62
	PLT (×10^9^/L)	988.6 ± 373.3	911.4 ± 267.8	00.46	0.22
Cardiovascular risk factors	Hypertension	8	24	1.61	0.2
	Diabetes	6	13	0.42	0.52
	Hyperlipidemia	5	8	0.15	0.69
	Smoking history	25	12	1.77	0.18

In ET patients, 4G4G genotype accounted for 25.8% (40/155), 4G5G genotype accounted for 38.7% (60/155), and 5G5G genotype accounted for 35.5% (55/155) (Table 1). Among them, the incidence of thrombotic events in 4G4G genotype was 54.5% (18/33), that in 4G5G genotype was 33.3% (11/33), that in 5G5G genotype was 12.2% (4/33), and that in PAI-1 genotype was 4G4G > 4G5G > 5G5G. The difference between the two was statistically significant (*P <* 0.05) (Table 2).

In ET patients, JAK2 V617F gene mutation rate was 58.7% (91/155), CALR gene mutation rate was 23.9% (37/155), MPL mutation rate was 7.1% (11/155), and triple negative mutation rate was 10.3% (16/155). In the JAK2 V617F + group, the incidence of thrombotic events (70.6%) is significantly higher than the non-thrombotic event rate (29.4%), and the difference is highly statistically significant (*P <* 0.001). The incidence of thrombotic events in the JAK2 V617F + group is significantly higher than that in the CALR+, MPL+, and Triple negative groups (all *P <* 0.05), confirming that this mutation type is an potential independent high-risk factor for thrombotic events in patients with ET (Table 3).

**Table 3:** Correlation analysis of driving gene mutation types and thrombotic events in patients with essential thrombocythemia

Driver gene mutation(*n*)	Thrombotic events (%)		χ^2^	*P*
	with	without		
JAK2 V617F+(91)	64(70.6%)	27(29.4%)	15.74	< 0.001
CALR+(37)	5(14.3%)	32(85.7%)	28.36	< 0.001
MPL+(11)	2(18.2%)	9(81.8%)	8.92	0.003
Triple negative(16)	3(18.8%)	13(81.2%)	11.45	< 0.001

In ET patients, the frequency of PAI-1 genotypes in JAK2 V617F gene mutation positive patients was 4G4G (64.7%) > 4G5G (23.5%) > 5G5G (11.8%). There is a highly statistically significant difference in the PAI-1 genotype distribution between the JAK2 V617F + mutation group and the general population (*P <* 0.05). There was no significant difference compared with JAK2 V617F gene mutation negative patients (*P >* 0.05), but there was significant difference compared with CALR gene mutation positive patients (*P <* 0.05) (Table 4).

**Table 4:** Correlation analysis between driving gene mutation type and 4G/5G polymorphism of PAI-1 gene in patients with essential thrombocythemia

Driver gene mutation(*n*)	PAI-1gene type(%)			χ^2^	*P*
	4G4G	4G5G	5G5G		
JAK2 V617F+(91)	59(64.7%)	21(23.5%)	11(11.8%)	32.69	< 0.001
CALR+(37)	11(28.6%)	16(42.8%)	10(28.6%)	22.37	< 0.001
MPL+(11)	8(72.7%)	2(18.2%)	1(9.1%)	1.24	0.54
Triple negative(16)	4(25%)	9(56.3%)	3(18.7%)	2.05	0.64

In ET patients, the plasma levels of PAI-1 in 4G4G group was significant higher than that in 4G5G and 5G5G groups (*P <* 0.05) (Table 5). Moreover, the plasma levels of PAI-1 in JAK2 V617F mutation group was higher than that in CALR, MPL and triple negative ET patients (*P <* 0.05) (Table 6).

**Table 5:** Correlation analysis between plasma levels of PAI-1 and 4G/5G polymorphism of PAI-1 gene in patients with essential thrombocythemia

PAI-1 genotype	Plasma levels of PAI-1 (ng/ml)		*t*	*P*
	With thrombotic events	Without thrombotic events		
4G4G	25.5	19.3	4.15	< 0.001
4G5G	20.3	17.2	2.01	0.048
5G5G	17.6	14.8	1.92	0.057

**Table 6:** Correlation analysis between plasma levels of PAI-1 and driving gene mutation type in patients with essential thrombocythemia

Driver gene mutation(*n*)	Plasma levels of PAI-1 (ng/ml)	*t*	*P*
JAK2 V617F+	27.3	--	--
CALR+	22.8	3.21	0.002
MPL+	22.5	3.45	< 0.001
Triple negative	21.7	4.02	< 0.001

At a median follow-up of 48 months, the recurrence rate of venous and arterial thrombosis in ET patients with 4G4G genotype and JAK2 V617F mutation positive and plasma levels of PAI-1 were higher than that in ET patients with 4G5G, 5G5G genotype and CALR, MPL mutated.

## Discussion

About 40–60% of patients with ET experience thrombotic events, and most of these are the arterial thrombosis, which is one of the important reasons for its main complications, high mortality and high disability rates [3–5]. Previous studies have confirmed that over 60 years old, JAK2 V617F gene mutation and cardiovascular risk factors such as diabetes, hyperlipidemia, hypertension and smoking were risk factors for ET thrombosis [7–9].

Plasminogen activator inhibitor-1 (PAI-1) belongs to the serine protease inhibitor superfamily, which can be produced by vascular endothelial cells, hepatocytes, megakaryocytes and other cells. PAI-1 is an important part of the fibrinolysis system in the body and a major regulator of plasminogen activation [12, 13]. The increase of PAI-1 activity in the body can inhibit normal fibrinolysis, making fibrin deposition easy to lead to thrombosis. There are multiple polymorphisms in the promoter of PAI-1 gene, among which there is a 4G/5G gene polymorphism with guanylic acid insertion or deletion at 675 bp upstream of the transcription start site. The genotypes are 4G4G, 4G5G and 5G5G respectively, which regulate the expression level of PAI-1. Several studies have confirmed that 4G allele is related to the increase of PAI-I gene transcription, that is, the plasma PAI-1 level of individuals with genotype 4G4G is the highest, followed by genotype 4G5G, The lowest plasma PAI-1 level was found in individuals with genotype 5G5G [14, 15]. The views that the level of PAI-1 activity and its gene 4G/5G polymorphism are closely related to arteriovenous thrombotic disease have been widely recognized [16, 17]. Tofler et al. prospectively investigated 3203 patients without cardiovascular disease, and found that the incidence of cardiovascular events was high in patients with high PAI-1 level [18]. Akhter et al. reported that the increase of PAI-1 activity and genotype 4G4G were associated with ischemic stroke in Indians of Indian descent [19]. Tang et al. also showed that genotype 4G4G is an independent risk factor for deep vein thrombosis [20].

Our results show that the incidence of thrombotic events in PAI-1 gene 4G4G is the highest, followed by genotype 4G5G, and genotype 5G5G has the lowest incidence of thrombotic events. The results of this study showed that the incidence of 4G4G genotype had high thrombotic events in ET patients, which was consistent with the previous reported [15]. It was confirmed that the 4G4G polymorphism of PAI-1 gene, which had a higher plasma levels of PAI-1, was associated with thrombotic events in patients with ET. Moreover, the occurrence of thrombotic events and non thrombotic events in ET patients had no significant difference in age, gender and blood cells.

The occurrence of thrombotic events is closely related to the prognosis of ET patients. Studies suggested that the incidence of thrombotic events in patients with JAK2 V617F mutation positive is higher than those with JAK2 V617F mutation negative and CALR mutation positive [11]. However, the exactly mechanism is still unclear. Considering that the occurrence of thrombotic events is related to PAI-1 gene polymorphism, that is, the incidence rate of thrombotic events of 4G4G genotype is high [21]. This study investigated whether there is a potential relationship between the mutation types of the Ph-MPN driver gene and PAI-1 polymorphism in the occurrence of thrombotic events. Among ET patients, those with JAK2 V617F gene mutation positive group has the highest incidence rate of PAI-1 genotype 4G4G, followed by genotype 4G5G, and the incidence rate of genotype 5G5G is the lowest, and there is a statistically significant difference compared with the incidence rate of PAI-1 genotypes of CALR gene mutation, indicating that JAK2 V617F positive mutation has a potential relationship with the incidence rate of PAI-1 genotype, and those with JAK2 V617F mutation has a high frequency of PAI-1 genotype 4G4G and plasma levels of PAI-1. This may be one of the reasons why the incidence of thrombotic events in JAK2 V617F mutation positive patients is higher than that in CALR mutation positive ET patients.

In conclusion, the occurrence of thrombotic events is an important complication of ET patients. Our study found that the 4G/5G polymorphism of PAI-1 gene in ET patients was related to the occurrence of thrombotic events. In ET patients, the frequency of 4G4G genotype and plasms levels of PAI-1 in JAK2 V617F mutation positive patients were significantly higher than that in CALR mutation positive ET patients. The 4G/5G gene polymorphism and plasma levels of PAI-1 and the positive mutation of JAK2V617F have potential value in predicting the risk factors for thrombotic events in ET patients. However, there are a lot of limitations in our study. First of all, retrospective study is classified as observational study and can only demonstrate the correlation between variables. Last but not least, it is limited by single center and small sample data. In clinical practice, ET patients with positive mutations of JAK2V617F can further detect the genotype polymorphism and plasma levels of PAI-1 to better guide the clinical prevention and treatment of these subsets.

## Data Availability

No datasets were generated or analysed during the current study.
